# Development and validation of apoptosis‐related signature and molecular subtype to improve prognosis prediction in osteosarcoma patients

**DOI:** 10.1002/jcla.24501

**Published:** 2022-05-16

**Authors:** Jinjiong Hong, Qun Li, Xiaofeng Wang, Jie Li, Wenquan Ding, Haoliang Hu, Lingfeng He

**Affiliations:** ^1^ Department of Hand Surgery, Department of Plastic Reconstructive Surgery Ningbo No. 6 Hospital Ningbo China; ^2^ Department of Otorhinolaryngology Head and Neck Surgery Ningbo Medical Center Lihuili Hospital Ningbo China; ^3^ Department of Orthopedics Ningbo Medical Center Lihuili Hospital Ningbo China

**Keywords:** apoptosis, immunotherapy, osteosarcoma, prognosis, signature

## Abstract

**Background:**

Previous evidence has shown that apoptosis performs integral functions in the tumorigenesis and development of various tumors. Therefore, this study aimed to establish a molecular subtype and prognostic signature based on apoptosis‐related genes (ARGs) to understand the molecular mechanisms and predict prognosis in patients with osteosarcoma.

**Methods:**

The GEO and TARGET databases were utilized to obtain the expression levels of ARGs and clinical information of osteosarcoma patients. Consensus clustering analysis was used to explore the different molecular subtypes based on ARGs. GO, KEGG, GSEA, ESTIMATE, and ssGSEA analyses were performed to examine the differences in biological functions and immune characteristics between the distinct molecular subtypes. Then, we constructed an ARG signature by LASSO analysis. The prognostic significance of the ARG signature in osteosarcoma was determined by Kaplan–Meier plotter, Cox regression, and nomogram analyses.

**Results:**

Two apoptosis‐related subtypes were identified. Cluster 1 had a better prognosis, higher immunogenicity, and immune cell infiltration, as well as a better response to immunotherapy than Cluster 2. We discovered that patients in the high‐risk cohort had a lower survival rate than those in the low‐risk cohort according to the ARG signature. Furthermore, Cox regression analysis confirmed that a high risk score independently acted as an unfavorable prognostic marker. Additionally, the nomogram combining risk scores with clinical characteristics can improve prediction efficiency.

**Conclusion:**

We demonstrated that patients suffering from osteosarcoma may be classified into two apoptosis‐related subtypes. Moreover, we developed an ARG prognostic signature to predict the prognosis status of osteosarcoma patients.

## Graphical abstract

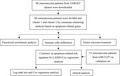

## 1 BACKGROUND

Osteosarcoma has been identified as the most prevalent type of primary bone malignancy in adolescents and children.^1^ Osteosarcoma usually occurs in the metaphysis, including the humerus, tibia, or femur, which leads to high mortality and disability rates, especially in patients with metastasis.^2^ Despite the significant advances in therapies, including immunotherapy, radiotherapy, chemotherapy, and differentiation therapy, the effectiveness in the treatment of patients with osteosarcoma has remained unsatisfactory in the past few decades due to genomic complexities and instability.^3^, ^4^, ^5^ Although there is an approximately 70% 5‐year survival rate for patients suffering from localized osteosarcoma, patients suffering from metastatic disease experience unfavorable overall survival (OS) rates of <20%.^6^ As a consequence, novel treatment targets must be explored, and biomarkers must be identified for the purpose of effectively stratifying patients and designing tailored therapy regimens for patients with osteosarcoma.

Apoptosis, also known as programmed cell death, is a main cellular process by which mammals eliminate DNA‐damaging cells and sustain tissue homeostatic control.^7^ There are two significant apoptosis pathways, namely, mitochondria‐mediated pathways (intrinsic pathway) and death receptor‐mediated pathways (extrinsic pathways).^8^, ^9^ Apoptosis is implicated in a variety of biological as well as pathological mechanisms, including the progression of tumors, oncogenesis, organ and tissue homeostasis, and embryonic growth.^10^, ^11^ Tumor cells have the capacity to escape programmed cell death, which could also increase invasiveness in the process of tumor growth, boost tumor angiogenesis, and accelerate cell proliferation.^12^, ^13^ Additionally, selective induction of apoptosis is one of the most effective anticancer therapies, including targeted therapy, radiotherapy, and chemotherapy.^14^ In view of the fact that large‐scale public databases comprising gene expression data as well as clinical information are now available, it has become feasible to create a highly accurate prognostic signature. In recent years, the development of apoptosis‐related gene (ARG) signatures for the risk assessment and prognosis prediction of cancers has become a research hotspot and has yielded excellent outcomes.^15^, ^16^ However, there is no clarifying ARG signature for predicting osteosarcoma patient prognosis.

To investigate the ARG molecular subtypes of osteosarcoma, we first obtained the ARG expression patterns and relevant clinical data of osteosarcoma patients from the TARGET database. Subsequently, utilizing ARGs from the TARGET cohort, we created a prognostic signature that was verified in the GEO cohort to enhance risk stratification as well as prognostic predictions in patients with osteosarcoma. Overall, the present research could aid in achieving an enhanced comprehension of the fundamental process as well as the evaluation of prognosis of osteosarcoma patients.

## 2 METHODS

### 2.1 Data collection

We downloaded the FPKM RNA‐sequencing data as well as relevant clinical data of 88 osteosarcoma patients (TARGET‐OS cohort) from the genomic data commons data portal (https://portal.gdc.cancer.gov/) as a training cohort. The clinical characteristics and mRNA expression data of 53 osteosarcoma patients in GSE21257 were acquired from the GEO database (https://www.ncbi.nlm.nih.gov/geo/) as a validation cohort. Table 1 illustrates the patients' clinical data.

**TABLE 1:** Clinical features of osteosarcoma patients in the present research

Clinical characteristics	Target		GSE21257	
	Number	Percent	Number	Percent
Gender				
Female	37	42.05	19	35.85
Male	50	56.82	34	64.15
Unknown	1	1.14	–	–
Age				
≤14	39	44.32	15	28.30
>14	48	54.55	38	71.70
Unknown	1	1.14	–	–
Ethnicity				
Caucasian	52	59.09	–	–
Asian	7	7.95	–	–
African descent	7	7.95	–	–
Unknown	22	25.00	–	–
Grade				
G1 + 2	7	7.95	29	54.72
G3 + 4	6	6.82	18	33.96
Unknown	75	85.23	6	11.32
Primary tumor site				
Leg	79	89.77	44	83.02
Arm	6	6.82	8	15.09
Pelvis	2	2.27	–	–
Unknown	1	1.14	1	1.89
Metastasis status				
Yes	22	25.00	14	26.42
No	65	73.86	39	73.58
Unknown	1	1.14	–	–
Survival status				
Dead	27	30.68	23	43.40
Alive	58	65.91	30	56.60
Unknown	3	3.41	–	–

### 2.2 Consensus clustering of osteosarcoma patients

We searched GSEA‐MSigDB (http://www.gsea‐msigdb.org/gsea) for ARGs by searching for “apoptosis” as a keyword, and 580 ARGs were identified (Table S1). To identify ARGs that were substantially correlated with the prognosis of osteosarcoma patients, we performed a univariate Cox proportional hazards regression analysis. For the purpose of performing subsequent analysis, 58 ARGs (Table S2) with a *p* value <0.05 were defined as prognosis‐related ARGs and subjected to further analysis. Subsequently, utilizing the “Consensus ClusterPlus” tool in R, these osteosarcoma samples were subjected to consensus clustering with a clustering factor (*k*) ranging from 2 to 9. The highest intragroup correlations and the lowest intergroup correlations were obtained when *k* = 2, demonstrating that the 88 osteosarcoma patients can be divided into two clusters, Cluster 1 and Cluster 2. The osteosarcoma patients in the two clusters were visualized in a two‐dimensional scatter plot after t‐distributed stochastic neighbor embedding (tSNE) and principal component analysis (PCA) dimensionality reduction.

### 2.3 Functional enrichment analysis

The threshold values for identifying differentially expressed genes (DEGs) across the two clusters were set as a false discovery rate (FDR) *p* < 0.05 and log2|fold change|>1. Gene Ontology (GO)^17^ and Kyoto Encyclopedia of Genes and Genomes (KEGG)^18^ pathway enrichment analyses were conducted according to these DEGs to examine the distribution of the biological functions between the two clusters. We also conducted gene set enrichment analysis (GSEA, version 4.0.1) between Clusters 1 and 2 for the purpose of examining the different enrichment of pathways.^19^ A FDR *p* < 0.05 was set to illustrate statistical significance.

### 2.4 Immune characteristics of the two apoptosis‐related clusters

First, the ESTIMATE algorithm^20^ was used to quantify the scores of the tumor microenvironment (TME) of each osteosarcoma patient, including estimate, immune scores, and stromal scores. Single‐sample gene set enrichment analysis (ssGSEA)^21^ was utilized to assess the enrichment scores of 16 distinct types of immune cells and the activities of 13 pathways correlated with immune function in each osteosarcoma sample. Then, we compared these scores between the two apoptosis‐related clusters. Ultimately, we examined the expression of immune checkpoint genes as well as human leukocyte antigen (HLA) genes between the two clusters to anticipate immunotherapy responsiveness.

### 2.5 Establishment and verification of an apoptosis‐related risk signature

In this protocol, we used the TARGET dataset as the training cohort for the purpose of developing the prognostic model. In addition, we obtained survival‐related ARGs in the univariate Cox analysis that had been preliminarily filtered to conduct the least absolute shrinkage and selection operator (LASSO) regression utilizing the glmnet R package,^22^ assisting in the identification of suitable factors with which to standardize the completed signature as well as prevent overfitting. In addition, utilizing the Survminer R package, we successfully extracted the prognostic risk score equation by performing a multivariate Cox regression analysis. According to the formula, each osteosarcoma patient's risk score can be derived as depicted below:

$$\text{Risk score}=\sum_{i=1}^{n}\text{coefi}\times \mathrm{ARG}\;\text{expression}$$

Coefi is the coefficient of ARG in the signature. Subsequently, the osteosarcoma patients were classified into low‐ and high‐risk cohorts based on the median value, which served as the threshold value in this study. The performance of the AGR signature was verified in 53 osteosarcoma patients who had survival data from the GSE21257 dataset as a validation set. The sva module in R was used to adjust all of the data from the two datasets. Time‐dependent 1‐, 3‐, and 5‐year receiver operating characteristic (ROC) curves, the Kaplan–Meier plotter with the log‐rank test, and multivariate and univariate Cox regression analyses were employed to examine the AGR signature's predictive power and accuracy. To determine whether our ARG signature had a superior predictive ability for osteosarcoma patients, we compared it with five published prognostic signatures related to glycolysis,^23^ immunity,^24^ hypoxia,^25^ ferroptosis,^26^ and metastasis.^27^ Moreover, a nomogram incorporating the risk model together with clinical data was developed to accurately predict the prognosis status of osteosarcoma patients. The prediction accuracy of the nomogram was confirmed utilizing calibration curves as well as time‐dependent 1‐, 3‐, and 5‐year ROC curves. Eventually, the chi‐square test was employed to examine the correlation between the risk score and the clinicopathological variables of osteosarcoma patients.

### 2.6 Statistical analysis

R software (version: 4.1.0) was employed to perform all statistical analyses and visualize the data. The chi‐square test or Wilcoxon signed‐rank test was utilized to conduct data comparisons between various cohorts. With the help of a Kaplan–Meier plotter with a log‐rank test, we created OS curves for the various cohorts. With respect to clinicopathological factors, multivariate and univariate Cox regression analyses were conducted to determine whether the risk score independently served as a prognostic predictor for patients suffering from osteosarcoma. A criterion of *p* < 0.05 was set to indicate statistical significance.

## 3 RESULTS

### 3.1 Apoptosis‐related genes distinguished osteosarcoma patients

**FIGURE 1:**
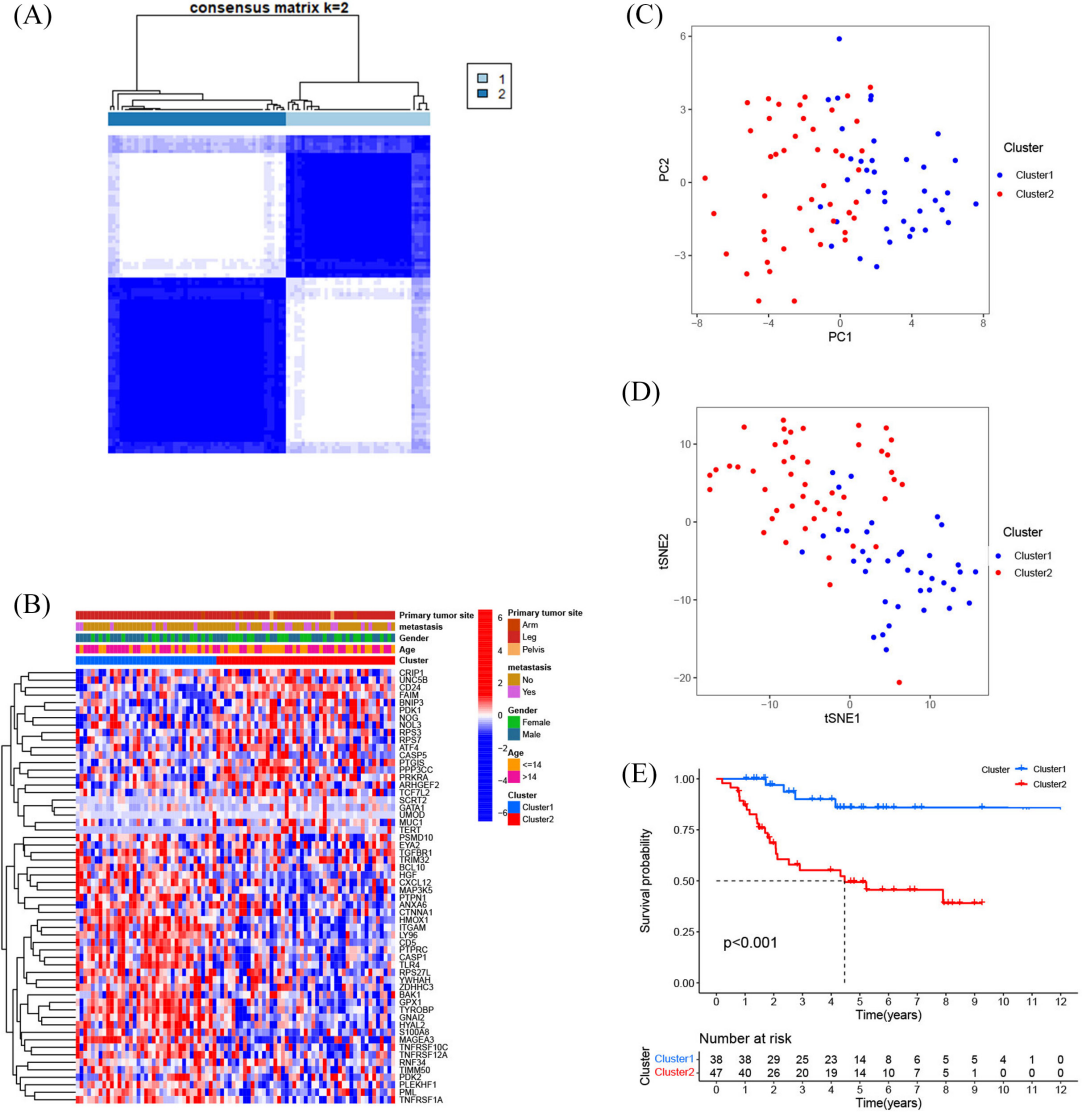
Consensus clustering analysis based on the apoptosis‐related genes. (A) Eighty‐eight osteosarcoma patients were classified into two clusters depending on the consensus clustering matrix and silhouette plot when *k* = 2. (B) The heatmap and clinical and pathological characteristics of the two clusters grouped according to their expression of apoptosis‐related genes. (C) Principal component analysis (PCA) of two clusters. (D) t‐distributed stochastic neighbor embedding (tSNE) analysis of two clusters. (E) Kaplan–Meier curves showing the overall survival of patients in the two clusters

A total of 58 ARGs associated with prognosis were discovered using univariate Cox regression analysis. According to consensus clustering analysis based on these prognosis‐related ARGs, 88 osteosarcoma patients could be well divided into Cluster 1 and Cluster 2 (Table S3; Figure 1A). A heat map (Figure 1B) showed the two clusters classified by 58 prognosis‐related ARGs. PCA (Figure 1C) and tSNE (Figure 1D) plotters based on ARG expression showed a clear distinction between the two clusters. Additionally, the Kaplan–Meier curve revealed that Cluster 2 had significantly worse OS than Cluster 1 (Figure 1E, *p* < 0.001).

### 3.2 Functional enrichment analysis between the two apoptosis‐related clusters

**FIGURE 2:**
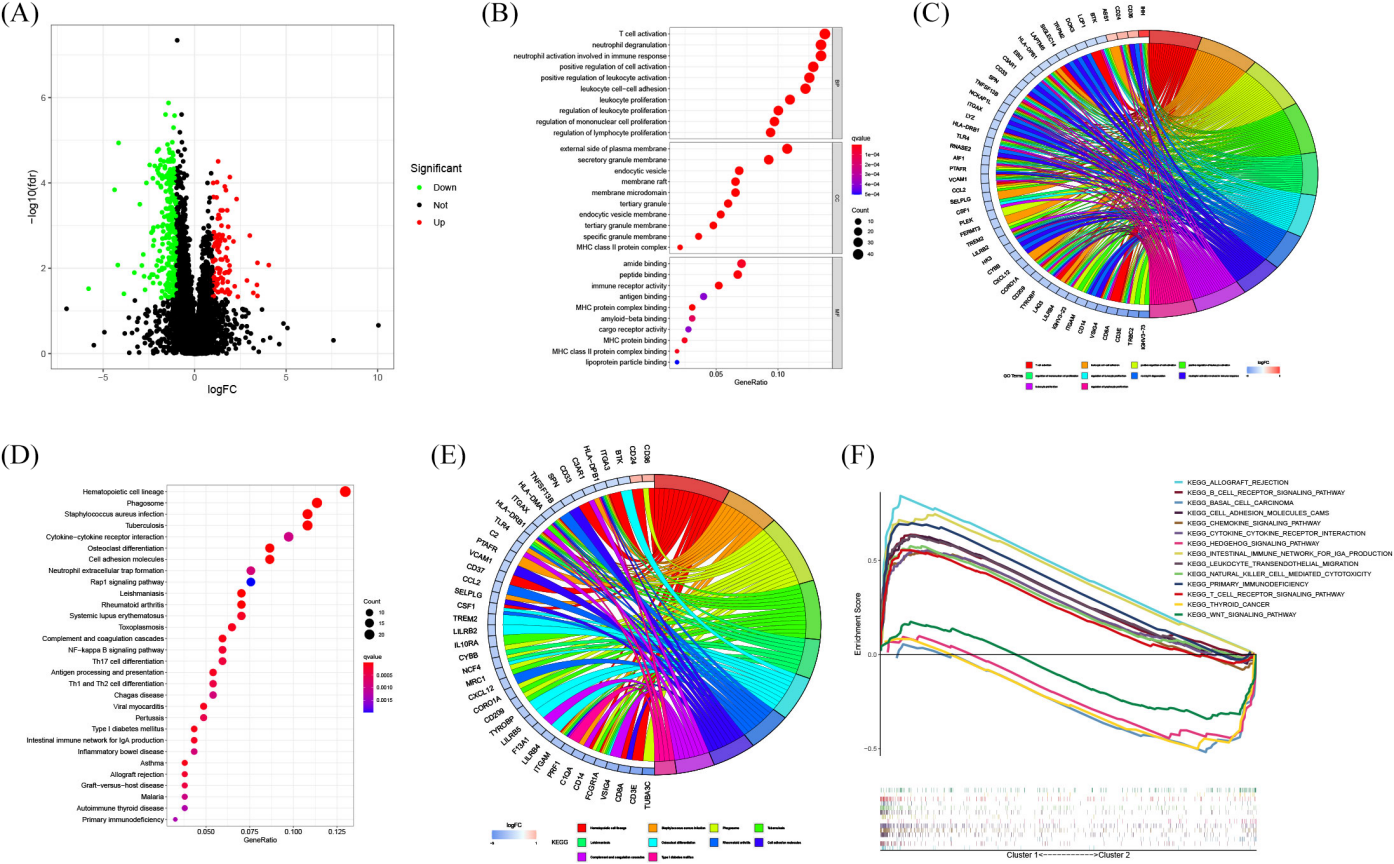
Functional enrichment analysis between the two clusters. (A) Volcano graph showing differentially expressed genes (DEGs) between the two clusters, where red dots denote upregulated genes, green dots denote downregulated genes, and black dots denote genes that did not differ. (B) Bubble plotter of GO enrichment analysis. (C) The correlations between DEGs and GO terms are shown in a chord plot. (D) Bubble plot of KEGG analysis results. (E) The correlation between DEGs and pathways is represented by a chord graph. (F) GSEA between the two clusters

To examine the possible biological functions across the two clusters, we next identified 389 DEGs (Figure 2A and Table S4) between Cluster 1 and Cluster 2 to perform GO‐enrichment analysis as well as KEGG pathway analysis. GO‐enrichment analysis (Figure 2B,C) showed that DEGs had considerable enrichment profiles in immune‐associated processes, such as the activation of T cells, modulation of mononuclear cell proliferation, immune receptor activity, and activation of neutrophils implicated in immunological responses. Then, through KEGG pathway analysis (Figure 2D,E), we found that the DEGs were predominantly associated with the Rap1 signaling pathway, cell adhesion molecules, hematopoietic cell lineage, the differentiation of osteoclast, and the interaction of cytokine‐cytokine receptor. A more in‐depth GSEA study revealed that there were differences in biological functions and pathways between the two clusters. Figure 2F clearly showed that some pathways correlated with the immune system were enriched in Cluster 1, such as the T‐cell receptor signaling pathway, primary immunodeficiency, cytotoxicity mediated by natural killer cells, the intestinal immune network for producing IgA, the interaction of cytokine–cytokine receptors, the B‐cell receptor signaling pathway and the chemokine signaling pathway. In contrast, Cluster 2 was related to cancer‐related pathways, such as the Wnt signaling pathway, Hedgehog signaling pathway, thyroid cancer, and basal cell carcinoma.

### 3.3 Analysis of immune infiltration characteristics

**FIGURE 3:**
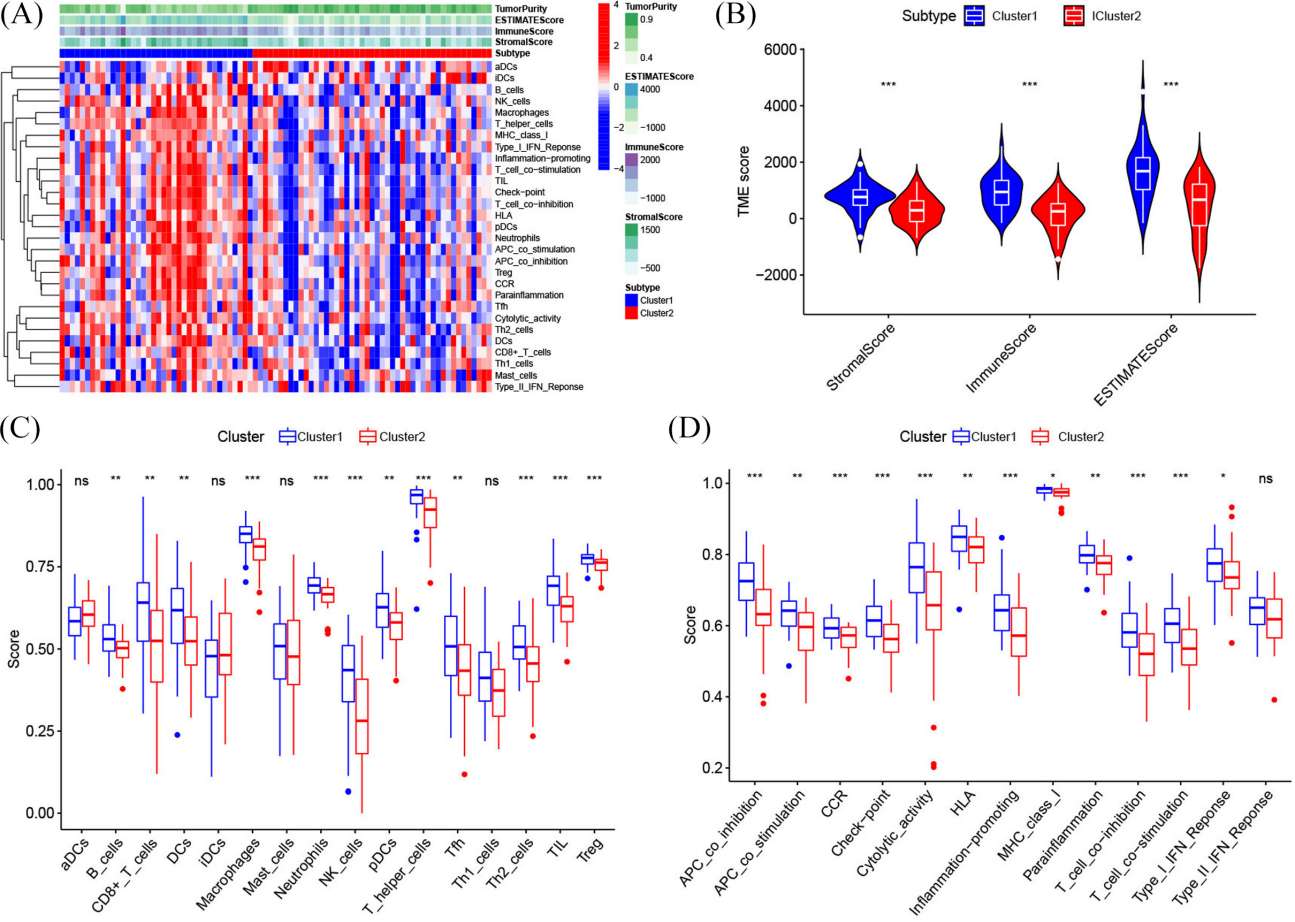
Tumor microenvironment (TME), immune cell infiltration, and pathways associated with immune cells between the two apoptosis‐related clusters. (A) Heatmap for the tumor microenvironment, immune‐related pathways, and immune cell infiltration between the two clusters. (B) Comparison of TME scores (estimate, immune, and stromal scores) between the two clusters. (C) A comparison of the enrichment scores for 16 different types of immune cells between the two clusters. (D) Thirteen pathways associated with immune cells between the two clusters. (ns denotes ‘no significance’, *p < 0.05, **p < 0.01, ***p < 0.001)

**FIGURE 4:**
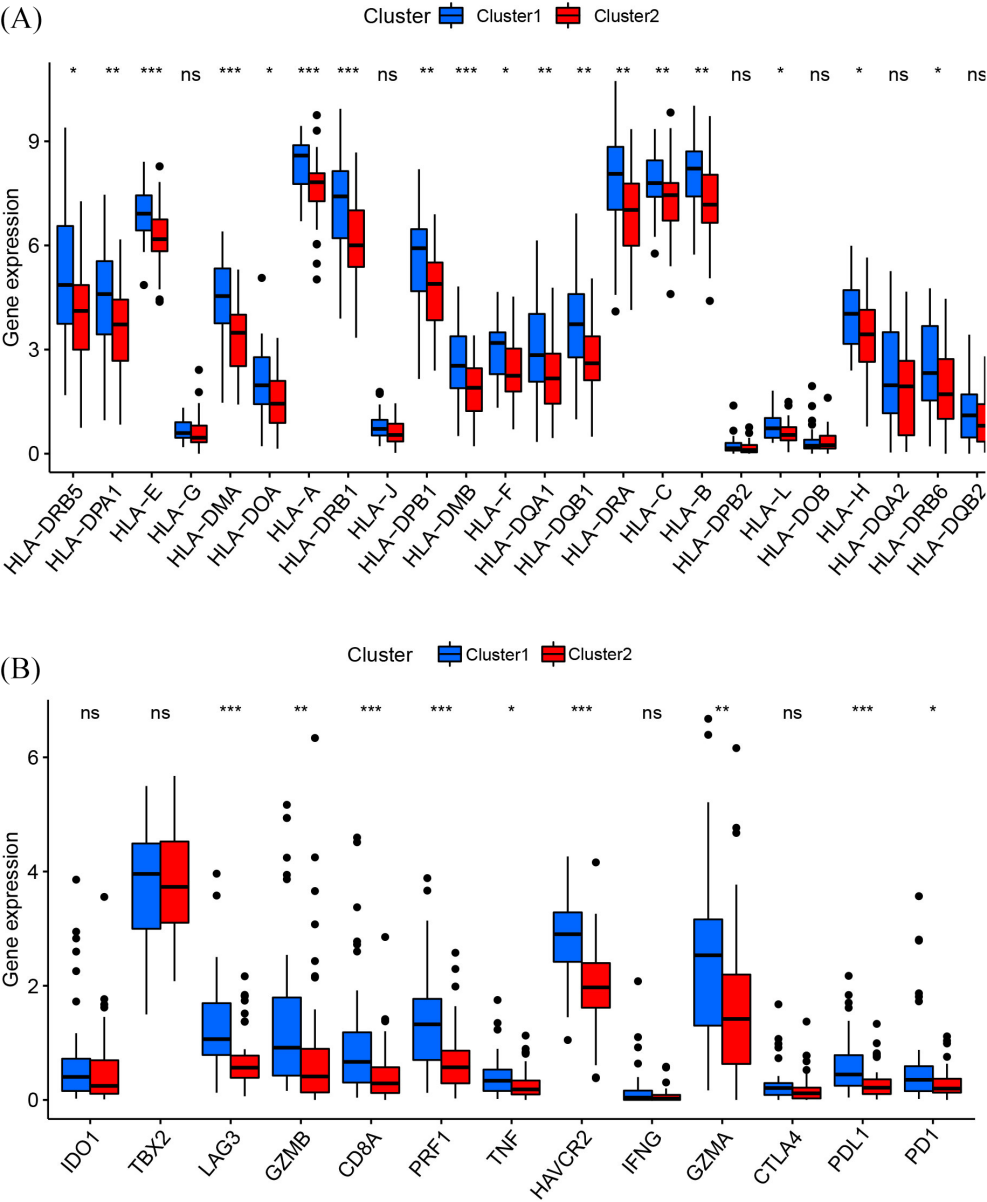
Correlation between apoptosis‐related clusters and immune‐related genes. (A) Differential expression of HLA‐related genes between the two clusters. (B) Differential expression of immune checkpoint‐related genes between the two clusters. (ns denotes ‘no significance’, *p < 0.05, **p < 0.01, ***p < 0.001)

We applied ESTIMATE and ssGSEA to explore the TME, immune‐related pathways, immune‐related functions, and immune cell infiltration (Figure 3A). TME analysis (Figure 3B) revealed that Cluster 1 showed statistically elevated ESTIMATE, immune, and stromal scores compared with those in Cluster 2. In addition, Cluster 1 had higher infiltration proportions of immune cells, such as dendritic cells, macrophage cells, CD8^+^ T cells, T helper cells, NK cells, and B cells, than Cluster 2 (Figure 3C). Analogously, the scores of immune‐related functions and immune‐related pathways in Cluster 1 were considerably elevated compared with those in Cluster 2 (Figure 3D). Immunotherapy has become an established pillar of anticancer treatment in recent years, which improves the prognosis for cancer patients. Therefore, we estimated the expression of genes associated with immune checkpoints and HLA in the two clusters of osteosarcoma patients. It was found that a large majority of HLA‐related genes (Figure 4A, *HLA‐DQB1*, *HLA‐DPA1*, *HLA‐E*, *HLA‐DMA*, *HLA‐DOA*, *HLA‐A*, *HLA‐DRB1*, *HLA‐DMB*, *HLA‐F*, *HLA‐DQA1*, *HLA‐DRA*, *HLA‐C*, *HLA‐B*, *HLA‐DRB5*, *HLA‐L*, *HLA‐H*, *HLA‐DPB1*, and *HLA‐DRB6*) and immune checkpoint‐related genes (Figure 4B, *LAG3*, *GZMB*, *CD8A*, *PRF1*, *TNF*, *HAVCR2*, *GZMA*, *PD1*, and *PDL1*) were significantly upregulated in Cluster 1 compared with Cluster 2.

### 3.4 Construction and evaluation of ARG signature

**FIGURE 5:**
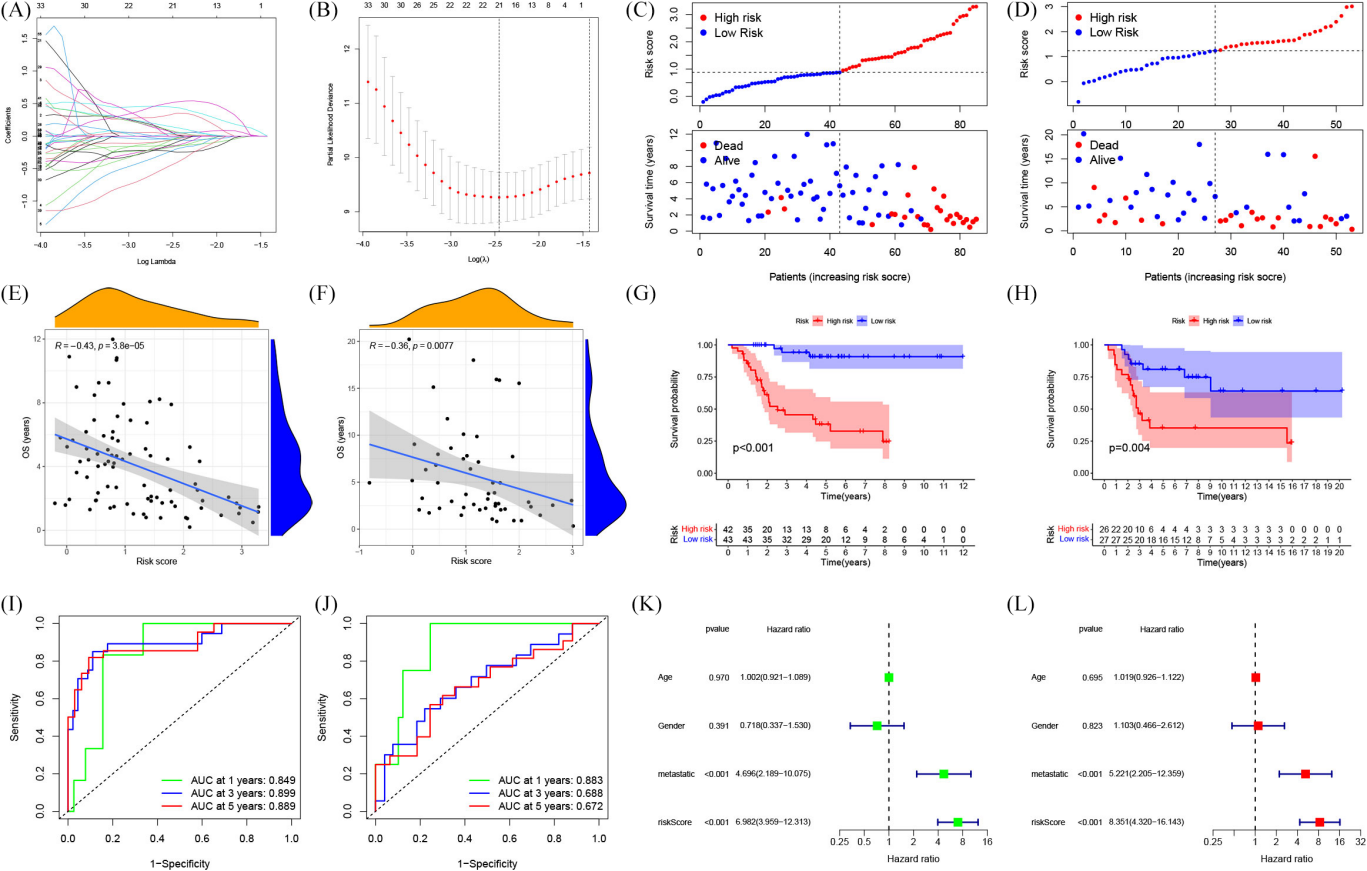
Development and validation of an apoptosis‐related prognostic signature. (A) Cross‐validation is performed in the LASSO regression analysis to optimize the variable screening process. (B) LASSO analysis with minimal lambda identified 21 potential genes. (C) In the TCGA cohort, the distribution of the risk score, as well as survival overview, were compared between the low‐ and high‐risk cohorts. (D) In the GEO dataset, the risk score distribution and survival overview were compared between the low‐ and high‐risk cohorts. (E) In the TCGA cohort, the correlation between survival duration and risk score was investigated. (F) In the GEO dataset, the correlation between survival duration and risk score was investigated. (G) In the TCGA dataset, the Kaplan–Meier curves of OS were compared between the low‐ and high‐risk cohorts. (G) In the GEO dataset, the Kaplan–Meier curves of OS were compared between the low‐ and high‐risk cohorts. (I) Patient data from the TCGA dataset were used to create time‐dependent 1‐, 3‐, and 5‐year ROC curves. (J) Patient data from the GEO dataset were used to create 1‐, 3‐, and 5‐year time‐dependent ROC curves. (K) The risk score and clinical parameters were evaluated by performing a univariate Cox regression analysis. (L) The risk score and clinical parameters were evaluated utilizing a multivariate Cox regression analysis

**FIGURE 6:**
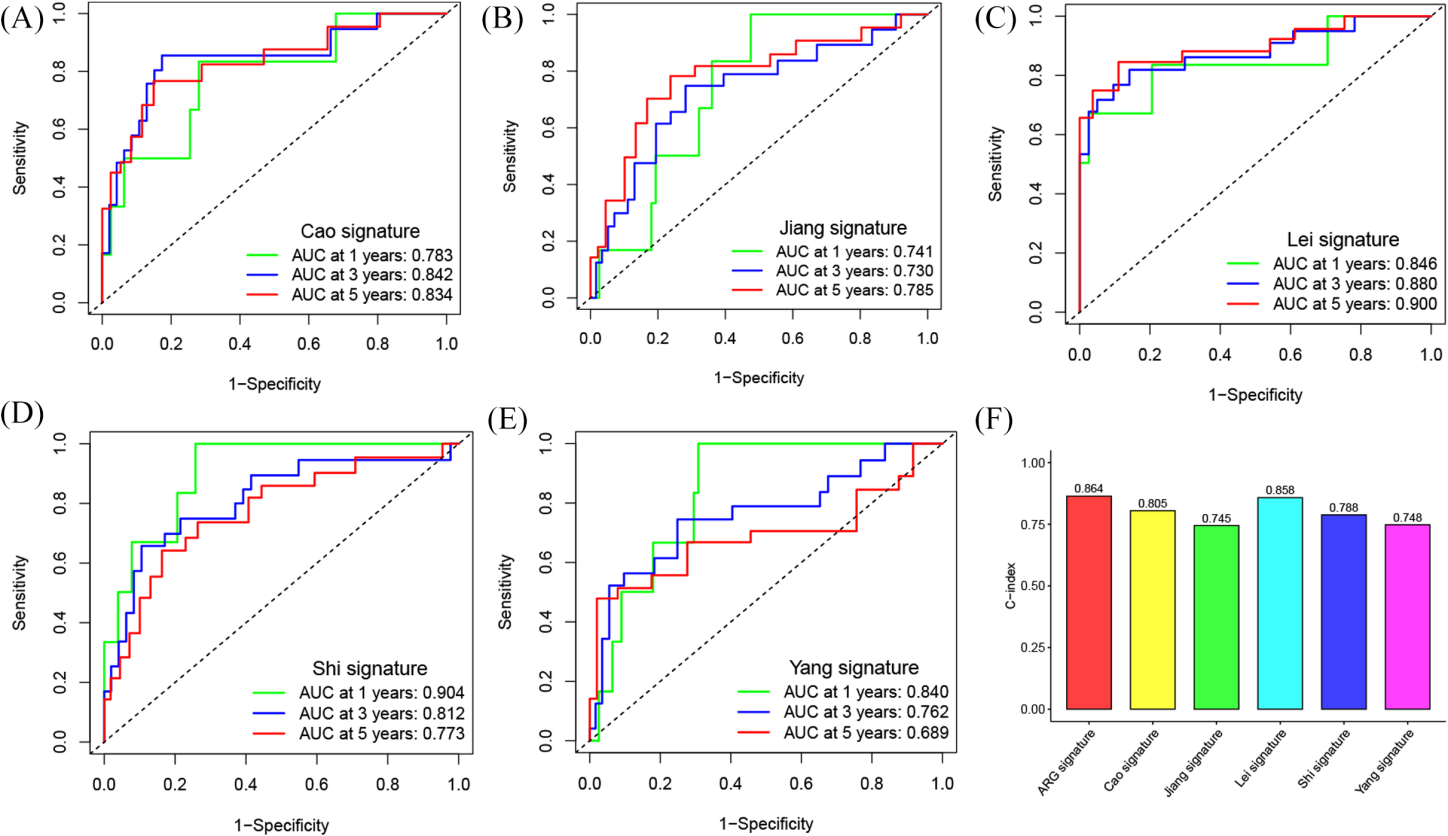
Comparison of the ARG signature with other signatures. (A) The ROC curves of an immune‐related gene signature.^24^ (B) The ROC curves of a hypoxia‐related gene signature.^25^ (C) The ROC curves of a ferroptosis‐related gene signature.^26^ (D) The ROC curves of a metastasis‐related gene signature.^27^ (E) The ROC curves of a glycolysis‐related gene signature.^23^ (I) C‐indices of the ARG signature and the other five signatures

**FIGURE 7:**
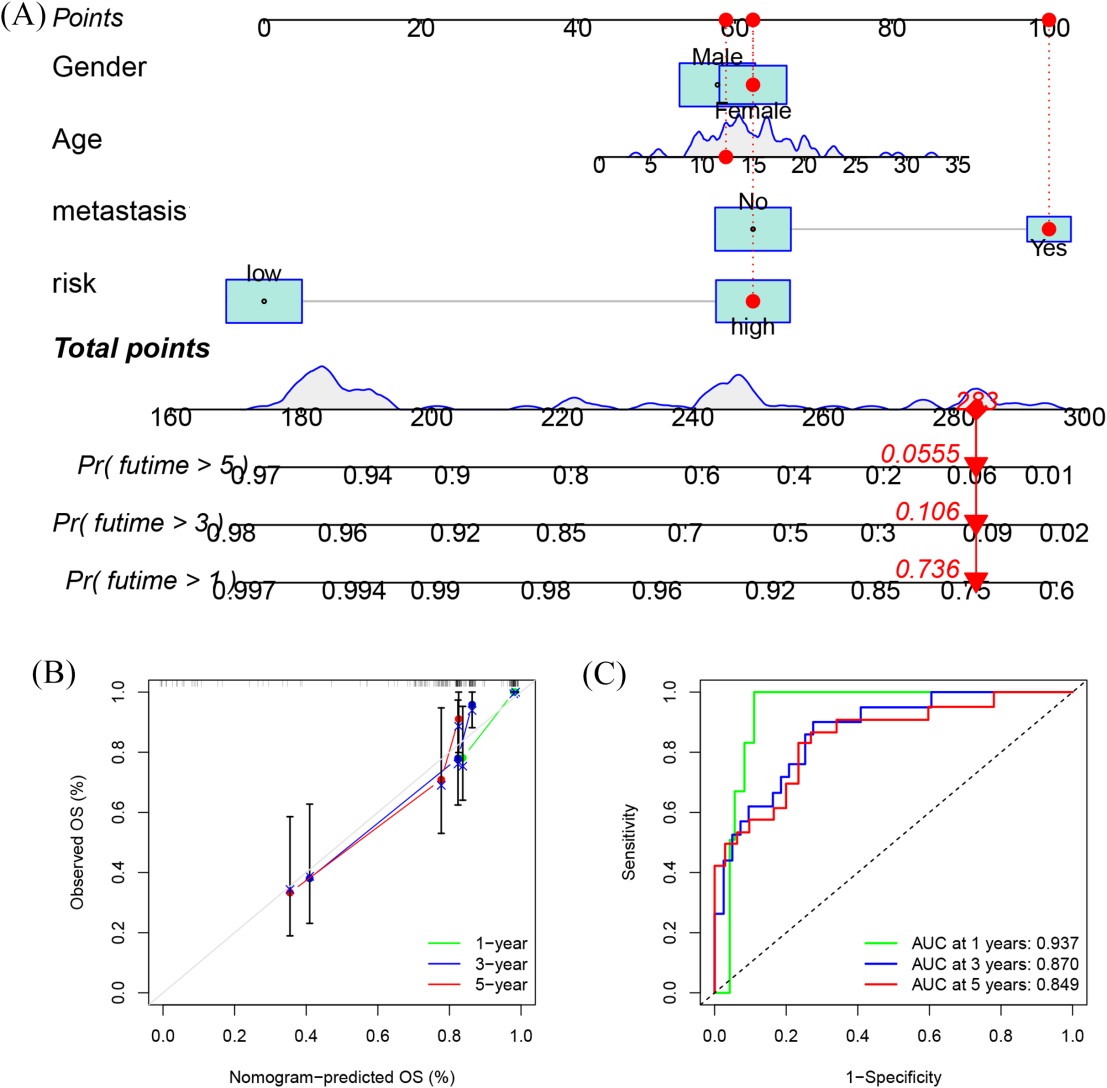
Development and assessment of a nomogram based on the ARG signature. (A) A nomogram for anticipating osteosarcoma patient prognoses according to the risk score and clinical characteristics. (B, C) Calibration chart (B) and 1‐, 3‐, and 5‐year ROC curves (C) used to validate the predictive performance of the nomogram

We used the TARGET cohort as the training set, whereas GSE21257 was the validation set. We constructed the ARG signature consisting of 21 ARGs on the training set using LASSO regression (Figure 5A,B). The coefficients of ARGs in the signature are shown in Table 2. The risk scores of patients suffering from osteosarcoma in the GSE21257 and TARGET cohorts were obtained, and the samples were classified into low‐ and high‐risk cohorts depending on their specific median scores achieved. The distribution landscapes of survival status and risk score for the samples in the TARGET and GSE21257 cohorts are depicted in Figure 5C,D, respectively. The Pearson correlation analysis demonstrated a substantially negative correlation between survival status and risk score in the GSE21257 cohort (Figure 5F, *R* = −0.36, *p* = 0.008) and TCGA cohort (Figure 5E, *R* = −0.43, *p* < 0.001). Kaplan–Meier curves of OS illustrated that the high‐risk cohort patients exhibited a substantially unfavorable prognosis in contrast to those in the low‐risk cohort in both the TCGA cohort (Figure 5G, *p* < 0.001) and GSE21257 cohort (Figure 5H, *p* = 0.004). Moreover, the time‐dependent ROC analysis of the TARGET cohort demonstrated that the 1‐, 3‐, and 5‐year AUC values for OS were 0.849, 0.899, and 0.889, respectively (Figure 5I), whereas in the GSE21257 cohort, the values were 0.883, 0.688, and 0.672, respectively (Figure 5J). Overall, the risk models in both datasets exhibited a high predictive capacity for the prognosis profile of osteosarcoma, according to the aforementioned findings. Subsequently, univariate (Figure 5K) and multivariate (Figure 5L) Cox regression analyses demonstrated that the risk score independently served as a prognostic marker for patients with osteosarcoma (HR: 8.351, 95% CI: 4.320–16.143, *p* < 0.001). To determine whether our ARG signature had a superior predictive performance, we calculated the AUC values and C‐index (Figure 6F) of five previously published signatures in the TARGET cohort. The AUC values (Figure 6A–E) of the five signatures for 1, 3, and 5 years were lower than those of our ARG signature. Meanwhile, our ARG signature had the highest C‐index at 0.864 (Figure 6F). It was also shown that an integrated nomogram based on the risk score and three clinical parameters may be employed to predict the prognoses of osteosarcoma patients (Figure 7A). The calibration curves regarding the nomogram (Figure 7B) for predicting 1‐, 3‐, and 5‐year OS were all close to the theoretical curve (45° line), showing that there was a high degree of agreement between the anticipated and observed results. The 1‐, 3‐, and 5‐year AUC values of the nomogram (Figure 7C) were 0.937, 0.870, and 0.849, respectively, demonstrating that the nomogram may be a viable prognostic model for the prognosis prediction of osteosarcoma patients.

**TABLE 2:** Coefficients of genes used in the signature

Gene	Coefficient
ARHGEF2	0.01366902
ATF4	0.3539663
BAK1	−0.030721347
BCL10	−0.194735056
BNIP3	0.440217961
CRIP1	0.019610751
EYA2	−0.022000565
MAGEA3	−0.146158789
PDK1	0.048849779
PDK2	−0.248592614
PLEKHF1	−0.043632439
PML	−0.157323635
PSMD10	−0.057405651
PTGIS	0.128940864
PTPN1	−0.143609316
RNF34	−0.406556251
RPS3	0.062098512
TERT	0.526581575
TGFBR1	−0.090918483
TRIM32	−0.027423747
UNC5B	0.047796417

### 3.5 Correlation between risk score and clinical parameters

**FIGURE 8:**
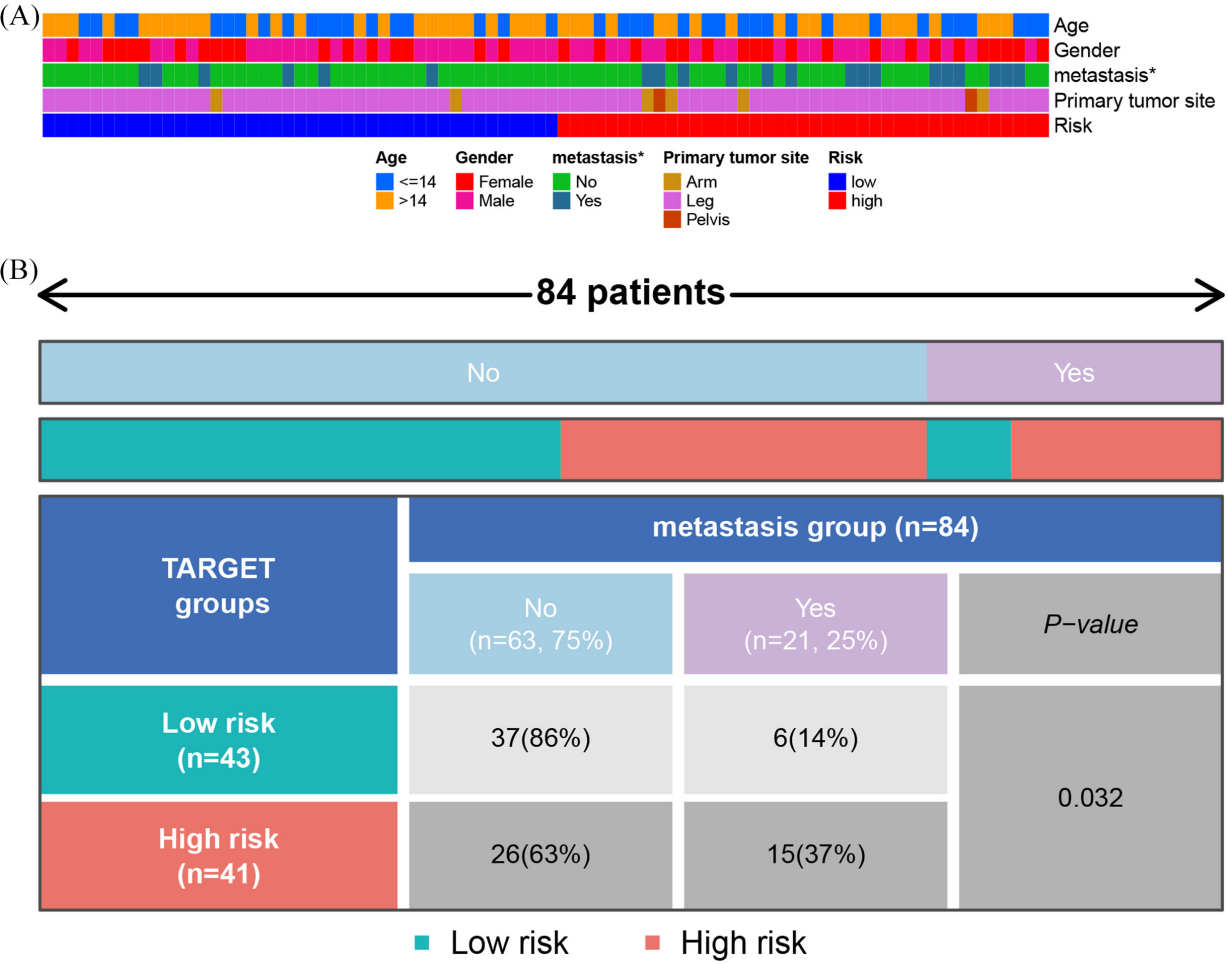
Association between clinical parameters and risk score. (A) Heatmap of the distribution of clinical characteristics between the low‐ and high‐risk cohorts. (B) The significant association between risk score and metastasis

Furthermore, the association between the risk scores and clinical variables was investigated. Based on clinical factors, we classified the patients into distinct groups. The heatmap (Figure 8A) showed that metastasis status was significantly different between the low‐ and high‐risk groups (*p* < 0.05), highlighting the fact that there were no statistically significant differences in any other clinical information between the two groups. As shown in Figure 8B, we classified the patients into four groups according to their risk scores and metastatic status. The chi‐square test demonstrated that osteosarcoma patients in the high‐risk cohort exhibited a greater metastatic rate than those in the low‐risk cohort (37% vs. 14%, *p* = 0.032).

## 4 DISCUSSION

Osteosarcoma is a prevalent malignancy that usually affects children as well as adolescents. Despite the fact that developments in surgical procedures and holistic treatments have enhanced the local control rate and improved the quality of life of osteosarcoma patients, the five‐year survival rate of osteosarcoma patients remains as low as 20% or less due to metastasis and recurrence.^6^, ^28^ The most effective method of improving the prognosis of these patients is to identify patients at high risk in a timely manner and to implement more comprehensive follow‐up plans as well as individualized/precision treatment. Thus, the identification of novel and specific biomarkers that can predict prognosis or monitor the clinical efficacy of osteosarcoma patients has good clinical value.

Recently, research reports have indicated that dysregulation of ARGs plays a critical role in the incidence and progression of various cancers by disrupting the balance between cell division and cell death,^29^, ^30^, ^31^ including osteosarcoma.^32^, ^33^, ^34^ For the purpose of smoothly conducting the present research, we used the GEO and TARGET datasets to obtain information on the expression of ARGs and osteosarcoma patients. By conducting consensus clustering analysis, we identified two apoptosis‐related molecular subtypes based on ARGs, named Cluster 1 and Cluster 2, and both PCA and tSNE plotters based on ARG expression showed a clear distinction between the two clusters. Kaplan–Meier analysis showed that Cluster 2 was correlated with a worse survival outcome, whereas Cluster 1 was correlated with a better survival outcome. Therefore, we explored the difference in biological function between the two subtypes. The GO enrichment and KEGG pathway analyses demonstrated that DEGs between the two clusters had a considerable enrichment landscape in immune‐related functions and pathways. Subsequent GSEA illustrated the enrichment of various immune‐related pathways in Cluster 1, while cancer‐related pathways were shown to be highly enriched in Cluster 2, indicating that Cluster 1 may have higher immunogenicity than Cluster 2. Growing evidence has shown that the increase in immunogenicity can activate the immune response, which contributes to a better prognosis for cancer patients.^35^, ^36^ To further assess immune infiltration characteristics between the two subtypes, we first utilized the ESTIMATE algorithm to explore the TME, which consists of stromal and tumor cells^37^ and performs an indispensable function in tumorigenesis,^38^ tumor progression,^39^ and metastasis.^40^ In the present research, we revealed that Cluster 1 exhibited a considerably greater level of stromal content and immune cell infiltration, as well as lower tumor purity, compared with Cluster 2. Furthermore, the results from ssGSEA confirmed that Cluster 1 exhibited elevated infiltration of several immune effector cells, including NK, B, and CD8^+^ T cells, which has the potential to trigger the identification process, ultimately resulting in the elimination of tumor cells. It has been shown that the high density of infiltrated CD8^+^ T lymphocytes is related to better patient outcomes in a variety of solid cancers.^41^, ^42^ Chen et al. discovered that a high density of infiltrated B cells in cholangiocarcinoma was correlated with improved prognosis.^43^ Furthermore, a growing body of evidence suggests that NK cells perform a critical function in antitumor immunity and are correlated with a favorable prognosis in certain cancers.^44^, ^45^, ^46^ From this point of view, higher infiltration levels of these immune effector cells in the TME may partially explain the better prognosis of Cluster 1 compared with Cluster 2 osteosarcoma patients.

Although immunotherapy is increasingly acknowledged as an effective and promising treatment for osteosarcoma,^47^, ^48^ there are currently no available biomarkers that can be used to stratify patients into different treatment options. Considering the difference in the immune landscape between the two clusters, we explored the expression of genes associated with immune checkpoints and HLA in the two clusters. Unsurprisingly, the results showed that the expression levels of most of these genes were related to immune checkpoints and that the expression level of HLA was significantly elevated in Cluster 1 compared with Cluster 2, especially for *PD1* and *PDL1*, implying that osteosarcoma patients in Cluster 1 had a high likelihood of benefiting from immune checkpoint inhibition treatment. Nevertheless, additional research is needed to determine the correlation between apoptosis‐related subtypes and the efficacy of immunotherapy in patients suffering from osteosarcoma.

We then established the prognostic ARG signature by LASSO regression. Our findings from the TARGET cohort, which were corroborated in the GSE21257 cohort, demonstrated that patients having a high risk score had a substantially shortened OS time compared with those having a low risk score, as shown in the risk model. The time‐dependent ROC curve and the AUCs demonstrated that this ARG signature had excellent predictive power in both the TARGET and GSE21257 cohorts. With the rapid development of bioinformatics, the latest research has reported several gene signatures associated with osteosarcoma prognosis from publicly available databases.^23^, ^25^, ^26^ Yang et al.^49^ discovered an immune‐related gene signature as a survival prognostic marker for patients suffering from osteosarcoma. Shi et al.^27^ defined genes differentially expressed between primary and metastatic osteosarcoma as metastasis‐related genes and used them to construct a novel metastasis‐related signature for the OS of patients with osteosarcoma. Compared with these signatures, we found that the ARG signature we generated showed better predictive efficacy in predicting osteosarcoma prognosis.

Furthermore, the findings obtained from the univariate and multivariate Cox regression analyses illustrated that the risk score may independently serve as a prognostic indicator for anticipating osteosarcoma patient prognoses. Notably, we discovered that integrating the risk score with clinical parameters to create a nomogram could much more accurately predict patient OS. Furthermore, the calibration and ROC curves of the nomogram illustrated that it could be an optimal prediction model.

Patients with advanced osteosarcoma are prone to distant metastases, particularly in the lungs, which are associated with poor clinical efficacy and prognosis.^50^ Thus, metastatic behavior remains the most crucial factor for doctors when stratifying risk and making treatment decisions for patients suffering from osteosarcoma.^51^ In the present research, we investigated the correlation between clinical parameters and signatures and illustrated that osteosarcoma patients within the high‐risk cohort presented elevated metastasis rates compared with the high‐risk cohort, indicating that the ARG signature could be useful for the prediction of metastasis in osteosarcoma.

The present research has a few limitations. First, given the low morbidity of osteosarcoma, the representative sample size employed in the present research was not large, and further confirmation in larger cohorts is needed. Second, the present research was focused solely on bioinformatic analysis, and additional experiments are required to confirm our findings. Third, our research is retrospective, and the ARG signature should be validated in large‐scale clinical trials and prospective studies. Finally, the biological functions of apoptosis‐related genes in the signature were not explored in osteosarcoma, and future studies are needed to investigate them.

In conclusion, we divided osteosarcoma patients into two subtypes based on ARGs. We then identified that Cluster 1 had higher immunogenicity and infiltration of immune cells, as well as a better response to immunotherapy, than Cluster 2. In addition, the ARG signature we developed can accurately predict the OS of osteosarcoma patients. Moreover, the nomogram we developed, which combines the ARG signature and clinical data, may exert a powerful prediction capacity for osteosarcoma patients. Additional investigation is necessary to validate the findings in the present research.

## AUTHOR CONTRIBUTIONS

JH and LH contributed to the design of the present research. QL, XW, JL, and WD collaborated to conduct a data analysis of the public databases. The data were analyzed by JH and HH. LH supervised the writing of the draft. The manuscript was reviewed by all the authors.

## CONFLICT OF INTEREST

All authors hereby state that there are no financial or other conflicts of interest associated with the present research.

## Supporting information


Table S1

Table S2

Table S3

Table S4

## Data Availability

The data that support the findings of this study are openly available from The Cancer Genome Atlas database (https://portal.gdc.cancer.gov/) and the Gene Expression Omnibus database (https://www.ncbi.nlm.nih.gov/geo).
